# The Effects of Ionizing Radiation on Gut Microbiota: What Can Animal Models Tell Us?—A Systematic Review

**DOI:** 10.3390/cimb45050249

**Published:** 2023-05-02

**Authors:** Ana Fernandes, Ana Oliveira, Raquel Soares, Pedro Barata

**Affiliations:** 1 Department Nuclear Medicine, Centro Hospitalar e Universitário de São João, E.P.E., 4200-319 Porto, Portugal; 2 i3S—Institute for Research and Innovation in Health, Universidade do Porto, 4200-135 Porto, Portugal; 3 Department of Biomedicine, Faculdade de Medicina, Universidade do Porto, 4200-319 Porto, Portugal; 4 Faculdade de Ciências da Saúde, Universidade Fernando Pessoa, 4200-150 Porto, Portugal; 5 Department of Pathology, Centro Hospitalar Universitário do Porto, 4099-001 Porto, Portugal

**Keywords:** microbiome, microbiota, intestinal microbiome, gut microbiota, ionizing radiation, radiotherapy, radiation effects

## Abstract

Background: The gut microbiota is relatively stable; however, various factors can precipitate an imbalance that is known to be associated with various diseases. We aimed to conduct a systematic literature review of studies reporting the effects of ionizing radiation on the composition, richness, and diversity of the gut microbiota of animals. Methods: A systematic literature search was performed in PubMed, EMBASE, and Cochrane library databases. The standard methodologies expected by Cochrane were utilized. Results: We identified 3531 non-duplicated records and selected twenty-nine studies after considering the defined inclusion criteria. The studies were found to be heterogeneous, with significant differences in the chosen populations, methodologies, and outcomes. Overall, we found evidence of an association between ionizing radiation exposure and dysbiosis, with a reduction of microbiota diversity and richness and alterations in the taxonomic composition. Although differences in taxonomic composition varied across studies, Proteobacteria, Verrucomicrobia, *Alistipes*, and *Akkermancia* most consistently reported to be relatively more abundant after ionizing radiation exposure, whereas Bacteroidetes, Firmicutes, and *Lactobacillus* were relatively reduced. Conclusions: This review highlights the effect of ionizing exposure on gut microbiota diversity, richness, and composition. It paves the way for further studies on human subjects regarding gastrointestinal side effects in patients submitted to treatments with ionizing radiation and the development of potential preventive, therapeutic approaches.

## 1. Introduction

The gut microbiota can be defined as the microorganisms (bacteria, viruses, archaea, and protists) that collectively inhabit the intestinal tract’s lumen and mucosal surface. The collection of all genomes of those microorganisms constitutes the intestinal microbiome [1,2].

The gut microbiota’s composition is established early in life, and it’s relatively stable over time. However, an imbalance of the gut microbiota’s composition, also known as dysbiosis, has been linked to a range of factors and diseases, including certain medical conditions such as inflammatory bowel disease, infections, or the overuse of antibiotics [2,3].

Ionizing radiation (IR) refers to energy capable of ionizing atoms or molecules by removing electrons from them. It results from radionuclides decay (unstable atoms) and may take the form of electromagnetic waves or particles [4,5,6,7].

Some of the molecular effects of ionizing radiation include DNA damage by breaking the strands or altering the bases, protein damage by altering the structure and function of proteins, by the production of reactive oxygen species (ROS), which can cause oxidative stress and damage to cells and tissues and by causing the cells to stop dividing and enter in a state of cell cycle arrest [4,5,6,7].

Overall, these molecular effects depend on the type and dose of radiation, as well as the sensitivity of the cells and tissues being exposed, and can lead to temporary cell dysfunction and, ultimately, lead to cell death or senescence [7,8].

The effects of ionizing radiation can be classified into two main categories: deterministic and stochastic effects. Deterministic effects are directly related to the level of radiation dose received. Stochastic effects, on the other hand, are those that are probabilistic and occur randomly without a minimum dose threshold [7]. Both effects are more common in tissues that are highly sensitive to radiation and that have a high rate of cell division, such as the skin, bone marrow and gastrointestinal tract [1,9,10].

Sources of ionizing radiation exposure include medical procedures, naturally occurring radioactive materials such as radon, cosmic radiation, industrial and occupational exposure, nuclear accidents and military activities [7,11,12].

The gut microbiota is a complex and diverse ecosystem of microorganisms, and understanding the effects of ionizing radiation on gut microbiota might provide insights into the causes of the gastrointestinal side effects of the treatments and lead to prophylactic/therapeutic attitudes. Ionizing radiation may induce alterations in the gut microbiota composition, richness, and diversity due to the modulation of microbial gene expression, induction of oxidative stress, and promotion of specific microbial species’ growth and suppression of others [4,5,6,7].

Most published studies evaluating the effects of ionizing radiation on the gut microbiota are in animal models. These studies allow perturbations in the gut microbiota to be studied in a controlled experimental setup and thus help assess the causality of the complex host-microbiota interactions and develop mechanistic hypotheses [13]. Hence, we sought to systematically review the existing evidence of the effects of ionizing radiation on gut microbiota in animal models.

The aim of this study was to undertake a systematic literature review to determine the effects of ionizing radiation on animals’ gut microbiota, namely in its composition, diversity, or richness/abundance.

## 2. Materials and Methods

### 2.1. Search Strategy and Selection Criteria

A systematic search was carried out using the following electronic databases: PubMed/MedLine (30 November 2022), EMBASE (31 December 2022), and Cochrane library (30 November 2022). Additional articles were identified through the reference list from the included articles and relevant reviews. To ensure that studies were not missed or wrongly excluded and that the search was comprehensive, we also searched gray literature, general search engines, and reference lists of included papers.

**Figure 1:**
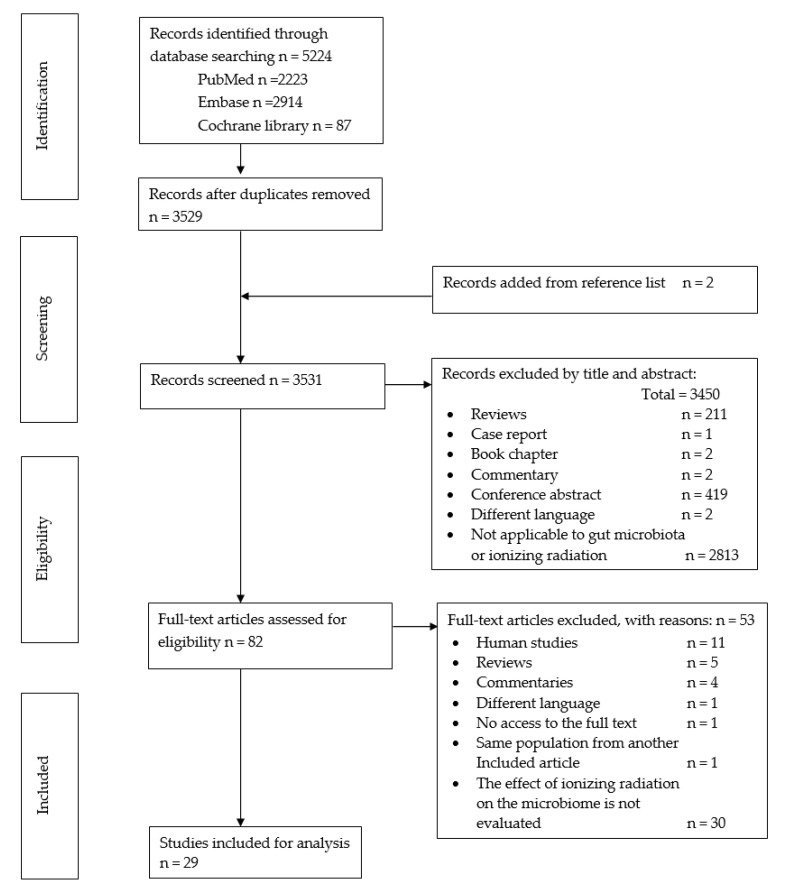
PRISMA flow chart search strategy.

This review was carried out following the Preferred Reporting Items for Systematic Reviews and Meta-Analyses (PRISMA) guidelines checklist. Additionally, the review protocol was registered on the International PROSPERO review database: PROSPERO 2020: CRD42020210951 (https://www.crd.york.ac.uk/prospero/display_record.php?ID=CRD42020210951) (accessed on 5 November 2022) (see Figure 1 for PRISMA diagram and Table 1 for search terms).

We first analyzed the effects of IR on the gut microbiota of humans [14]. During our search, we found that most studies were performed in animal models. In addition to anatomic and physiological differences, human and animal studies present significant methodological differences. Therefore, we consider it relevant to focus this review on animal studies.

The PROSPERO database and Cochrane Library search revealed no similar systematic reviews.

All selected citations were exported from the databases to the reference management software EndNote X20 (Thompson Reuters, New York, NY, USA), and duplicates were excluded.

### 2.2. Inclusion and Exclusion Criteria

Inclusion criteria were defined using the following components: patient population (P): animals exposed to radiation; exposure of interest (I): ionizing radiation; comparator (C): before and after exposure of the same subject or with controls; outcome (O): changes in the gut microbiome following exposure to radiation and the study design (S) of interest: interventional studies, prospective and retrospective observational cohort studies. The exclusion criteria were other types of studies (e.g., case-report, reviews); human or in vitro studies; and no relevant outcomes reported.

### 2.3. Study Selection and Data Extraction

All relevant peer-review journal articles in English, Portuguese, and Spanish, indexed until December 2022, were identified. A combination of search terms was used: microbiome, gut microbiota, radiotherapy, ionizing radiation, and 16S rRNA (Table 1, Table 2 and Table 3). The last search was performed on 31 December 2022 by two authors (AF and PB).

According to the defined inclusion and exclusion criteria, relevant studies were independently screened by two reviewers (AF and PB) based on title and abstract. All decisions were recorded on a spreadsheet.

All studies that did not fulfill the defined PICOS characteristics, conference papers, abstracts, and articles from which we could not obtain the full text were excluded.

Full-text papers of all eligible studies were obtained, and the two reviewers independently screened and selected papers a second time.

A tabular summary was developed with the following variables extracted from each eligible study: First author name, date of publication, study design, number of patients and controls, radiation exposure characteristics, type, number, and timepoint of samples, and the most relevant findings (Table 2 and Table 3).

### 2.4. Risk of Bias in Individual Studies

Two reviewers (AF and PB) assessed the risk of bias in each study independently, with disagreements resolved by consensus. The risk of bias was assessed as described in the Cochrane Handbook [15] by recording the methodology used.

The included studies’ quality was assessed by using the risk of bias tool from the Systematic Review Centre for Laboratory Animal Experimentation (SYRCLE) for animal studies [16]. Categories for the investigation of quality were as follows: (1) sequence generation; (2) baseline characteristics; (3) allocation concealment; (4) random housing; (5) blinding for the performance bias; (6) random outcome assessment; (7) blinding for the detection bias; (8) incomplete outcome data; (9) selective outcome data; and (10) other sources of bias. Assessment of each category was divided into high, low, or unclear risk of bias.

**Table 2:** Summary of study characteristics, demographics, radiation type, sample collection and analysis, and main findings of the eligible studies with mice.

Author, Year / Study Design	Participants / N Irradiated	Microbiome Assessment Method / Type of Sample / Number of Samples	Main Findings
	Type of Radiation		
Li Y, 2020 [17] / Interventional	Mice C57BL/6J Male and female 8 weeks N = 5	16s rRNA V4 region / Illumina Miseq / Fecal N = 3 Before irradiation, and 6 days and 12 days after irradiation	Diversity/richness α-diversity—decreased. β-diversity—changed Chao1 and ACE diversity index—unaltered Composition *Desulfovibrionaceae*—significant increase in the relative frequency *Lactobacillaceae*—decreased *Anaerotruncus*, *Coprococcus_1* and *Erysipelatoclostridium*—increased
	γ-ray TAI Single dose of 12 Gy or 15 Gy TBI Single dose of 4 or 7 Gy		
Yamanouchi K, 2018 [18] / Interventional	Mice C57BL/6Njcl Female 8 weeks N = 6	DNA Primer PCR / NucleoSpin^®^ DNA Stool / Fecal N = 8 before irradiation, at 1, 2, 6, 12, 24, 48 and 72 h after irradiation	Composition *Bifidobacterium*—2 Gy—decreased. 4 Gy—increased.∙ *Lactobacillus*—2 and 4 Gy decreased from 6 h to 12 h after irradiation and then recovered up to the baseline level in both groups
	X-ray TBI Single dose of 2 Gy and 4 Gy		
Goudarzi M, 2016 [19] / Interventional	Mice C57BL/6J Male 8 weeks N = 14 7 control	16s rRNA V4 region / Illumina HiSeq 2500 QIIME version 1.8.0 Phyloseq packages / Fecal N =3 0 and 5 Gy groups (1 day before and 3 and 30 days post-irradiation) N = 2 12 Gy group (1 day before and 3 days post-irradiation)	Diversity/richness Chao1 and Shannon—decreased on day 3 after 5 Gy irradiation, but recovered by the end of the 30-day study. α-diversity—12 Gy—unaltered. Day 3 after 5 and 12 Gy irradiation, 90 and 82 OTUs, respectively, were differentially abundant compared to day 1 before irradiation, and 12 of these OTUs were in common. Day 30 after 5 Gy irradiation, 91 OTUs had significantly different abundance compared to the pre-irradiation. 24 of the differential OTUs were in common on day 3 and 30 Composition Firmicutes—decreased Verrucomicrobia—increased and recovered by day 30 *Lachnospiraceae*, *Ruminococcaceae* and *Clostridiaceae* decreased after 5 and 12 Gy irradiation. *Lactobacillaceae*, *Staphylococcaceae*, *Bacteroidaceae* and two members of the *Ruminococcaceae* family increased. *Ruminococcus gnavus*, decreased significantly *Eubacterium biforme* decreased
	X-rays TBI Single dose 5 or 12 Gy		
Cui M, 2017 [20] / Interventional	Mice C57BL/6 Male and Female 6-8 weeks / N = 4	16s rRNA V4 region / Illumina Hiseq Uparse / Fecal N = 2 Days 5 and 10 postirradiation	Diversity Diversity of enteric bacteria altered in males but not in females Composition Composition of enteric bacteria altered in males but not in females
	γ-rays TBI Single dose 6.5 Gy		
Sittipo P, 2020 [21] / Interventional	Mice C57BL/6L Male 8 weeks / N = 10	16s rRNA V4-V5 regions / Qubit 2.0 Fluorometer and 2100 Bioanalyzer Ion Torrent PGM platform QIIME v1.9.1 and Microbiome Helper package / Fecal N = 4: -1 day before irradiation -3 (D1, D3 and D10)	Diversity/richness Diversity—decreased Richness—decreased Composition Firmicutes—Decreased at D1; recovered at later time points D3 and D10. *Ratio* Firmicutes/Bacteroidetes decreased Irradiation-resistant bacteria: *Ruminococcaceae*; *Lachnospiraceae*; *Clostridiaceae* *Lactobacillus* dramatically reduced at D1, recovered at D3 and D10
	γ-rays TBI Single dose 6 Gy		
Gerassy-Vainberg S, 2018 [22] / Interventional	Mice C57BL/6J Female 6-8 weeks / N = 23 Control n = 22	16S rRNA / QIIME V 1.8.0 Illumina Miseq platform Roche 454 Pyrosequencing / Fecal N = 3 1 week before 2 and 6 weeks post radiation	Composition Significant shift in post-radiation gut microbial composition. The most significant shift in microbial composition was observed 6 weeks post-radiation. Compared with controls: Proteobacteria (*Suterella* spp.)—significant change in abundance. Verucomicrobia (*Akkermansia* spp)—significant change in abundance. Firmicutes—decreased
	γ-rays 4 fractions of 550 cGy Localized internal rectal radiation		
Liu X, 2019 [23] / Interventional	Mice BALB/c / Male 8 weeks / 4 groups: Control; low-dose 6 mice sampled at each timepoint N = 24	16s rRNA V4 / Qiagen Mini Kit Qubit 2.0 fluorometer Illumina Hiseq / Fecal N = 4 Before 7, 21 and 35 Postirradiation	Diversity/richness Species number decreased significantly in all groups Shannon index decreased significantly in all groups Beta diversity in the LT10 group was significantly different. Simpson index increased significantly Number of OTUs decreased significantly. However, no difference was found among groups LT1, LT5 and LT10. Composition Bacteroidetes—significantly decreased in the LT10 Proteobacteria abundance increased in the LT10 *Bacteroida* significantly decreased in LT10 *Clostridia* increased in LT10 *Clostridiales* increased in the LT10 group *Porphyromonadaceae*—significantly decreased in the LT10 group *Lachnospiraceae* and *Ruminococcaceae* increased in the LT10 group *Clostridium*, *Helicobacter,* and *Oscilibacter* significantly increased *Bacteroides* decreased in the LT10 group *Barnesiella* decreased in the LT10 group
	γ-rays TBI group 1–0.5 Gy ×1 dose group 5–0.1 Gy per dose ×5 doses group 10–0.0 Gy per dose ×10 doses		
Johnson LB, 2004 [24] / Interventional	Mice C57/Bl6 / N = 30 6/per time point controls	Anaerobic vs. Anaerobic / Viable counts / Tissue samples from the irradiated small intestine N = 5 2, 6, 16, 24 and 48 h	Composition *Enterobacteriaceae*—decreased 2 h after; significantly decreased sixteen hours after; no significant differences 24 h after. *Lactobacillus*—decreased 2 h after and significant decrease after sixteen hours. There were no significant differences 24 h after. Anaerobic counts—decreased 2 h after. There were no significant differences 24 h after. Aerobic counts—Significantly decreased 2 h and sixteen hours after.
	X-rays / Single dose 19 Gy		
Lu L, 2019 [25] / Interventional	Mice C57BL/6 Female 4 to 5 week old / N = 18 6/groups RT only; Control; RT + PC	16s rRNA V3 and V4 Illumina Hiseq platform / Fecal N = 1 24 h after	Diversity/richness α- diversity—decreased Chao1 and Shannon indexes decreased without significance Composition Proteobacteria—increased Firmicutes—decreased *Lachnospiraceae*—decreased *Prevotellacea*—unchanged *Escherichia-Shigella*—increased. *Roseburia*, *Alistipes* and *Ruminococcaaceae*—decreased *Bifidobacter* and *Lactobacillus*—unchanged
	X-rays TAI Single dose 12 Gy		
Casero D, 2017 [26] / Interventional	Mice C57BL/6 Male 6 months / N = 30 10 controls	16S rRNA V4 region / Illumina HiSeq 2500 QIIME / Fecal N = 2 after 10 and 30 days	Diversity/richness Bacterial diversity—decreased α-diversity significantly different Diversity marked increase at 30 days for mice radiated at 0.1 Gy Beta diversity—significant differences regardless of dose levels Composition Actinobacteria (*Bifidobacterium* genus and *Adlercreutzia* unclassified species), Bacteroidetes (S24–7 unclassified species) and Firmicutes (unclassified *Lactobacillus* and *Clostridiaceae* species) decreased *Bifidobacteriales*, *Coriobacteriales*, *Verrucomicrobiales*, *Lactobacillales* significant perturbation *Bifidobacterium*—decreased *Roseburia*—increased Verrucomicrobia species—increased *Adlercreutzia* unclassified species; S24–7 unclassified species; unclassified Lactobacillus and *Clostridiaceae* species—decreased *Mollicutes* species (Tenericutes phylum) extinguish *Akkermansia muciniphila*—increased *Ruminococcus gnavus* marked expansion *Peptococcaceae* species, including the abundant rc4–4, reached normal levels at 30 days after a decline at 10 days in most animals exposed to 0.1 Gy Higher sensitivity of the gut ecosystem to lower doses—0.1 and 0.25 Gy as compared to the highest dose—1 Gy
	TBI ^16^O (600 MeV/n) at 0.1, 0.25, and 1 Gy		
Kim YS, 2015 [27] / Interventional	Mice C57BL/6 Male 8–10 week old / N = 3 Control = 2	16S rRNA UltraClean^®^ Fecal DNA Isolation Kit / Illumina MiSeq // Fecal (small and large intestine) / N = 1 3 days after irradiation.	Composition Verrucomicrobia identified in the irradiated samples but not in the control samples. Proteobacteria—increased Actinobacteria—decreased Bacteroidetes increased Firmicutes increased in the small intestine and decreased in the large Large intestine: *Alistipes*, *Lactobacillus* and *Akkermansia* increased *Barnesiella*, *Prevotella*, *Bacteroides*, *Oscillibacter*, *Pseudoflavonifractor* and *Mucispirillum*—decresaed *Lactobacillus*, *Prevotella* and *Akkermansia* abundances were altered dramatically (>5%). Small intestine: *Turicibacter*, *Corynebacterium*, *Alistipes*, *Lactobacillus* and *Mucisprillum* irradiation-susceptible microorganisms; *Corynebacterium*—increased *Alistipes*—marked decrease
	γ-rays TBI Single dose 8 Gy		
Wang M, 2020 [28] / Interventional	Mice C57BL/6J Male 8–10 weeks / N = 70 Intestinal group/Survival group Hematopoietic experiments	16s rRNA V3-V4 / Illumina MiSeq QIIME / Feces removed from the rectum (Small intestines were taken out after three days of irradiation) N = 1 3 days after IR	Diversity/richness Chao1 index, Simpson index and Shannon index—no significant difference. Composition Bacteroidetes—decreased Proteobacteria—increased Bacteroidetes/Firmicutes *ratio* decreased *Bacteroides*, *Alistipes*, *Parabacteroides*, *Ruminococcaceae_UCG-014*—increased *Lactobacillus*, *Prevotellaceae_UCG-001*—decreased
	γ-ray TBI 9.0 Gy—intestinal group 10.0 Gy—survival group 4.0 Gy—hematopoietic group		
Zhao Z, 2020 [29] / Interventional	Mice C57BL/6 Male 8–10 weeks / N = 4/3 4 pre radiation 3 post radiation	16S rRNA V4 region / QIIME (v 1.8) PANDAseq (version 2.9) / Fecal Terminal ileum and cecum 3 months after	Diversity/richness Number of OTUs detected and Chao index—decreased OTUs 290 common in the pre- and post-radiation/181 only pre/37 only post Simpson diversity index increased alpha- and beta-diversity decreased Composition Proteobacteria—increased Verrucomicrobia—decreased Bacteroidetes—decreased Firmicutes—decreased Actinobacteria—decreased
	γ-rays TAI Single dose of 10 Gy		
Wang W, 2020 [30] / Interventional	Mice / N = 18 Controls = 6	16s rRNA / Illumina MiSeq qRT-PCR / Fecal samples directly collected from the lower segment of the colon N = 2 Days 7 and 30 after irradiation	Diversity/richness α-diversity— no significant differences The gut microbiome did not change significantly at 7 days after IR; however, at 30 day after IR, obvious changes in bacteria were observed. LEfSe showed that no different bacteria were found between the normal control and IR groups at 7 days Composition Firmicutes—decreased *Verrucomicrobiaceae*—increased *Bacteroidales*_S24-7_group—increased uncultured_ bacterium_f_Bacteroidales_S24-7_group—increased *Lactobacillus*—control 14.67% vs. 7 days 17.19% vs. 30 days 6.67% *Ruminococcaccae_UCG014*—control 7.61% vs. 7 days 9.69% vs. 30 days 6.36% *Lachnospiraceae_NK4A136*—decreased *Prevotellaceae_UCG-001*—decreased *Akkermansia*—increased *Bacteroides*—control 5.25% vs. 7 days 4.44% vs. 30 days 4.8% uncultured_bacterium_f_*Lachnospiraceae*—decreased *Alistipes*—control 2.5% vs. 7 days 3.63% vs. 30 days 2.43% *Alloprevotella*—decreased *Eubacterium*_coprostanoligenes_group—decreased
	TBI		
Zhao Y, 2019 [31] / Interventional	Mice C57BL/6J Male 8–12 weeks / N = 5	16s rRNA / Illumina Hiseq / Fecal Fresh from rectum 10 month after	Diversity/richness Chao1 index—No alteration 10 months after *p* = 0.64 Weight_unifrac index—no alteration 10 months after *p* = 0.12 Shannon index—decreased clostridiaceae_1—increased *p* = 0.042 Composition *Quinella*—decreased significantly; *p* = 0.029 *Streptococcus_gallolyticus*—decreased significantly *p* = 0.034 Relative abundance, compared with controls: *Bacteroidia*—increased *Clostridia*—decreased *Erysipelotrichia*—increased *Betaproteobacteria*—increased Unidentified_*Saccharibacteria*—decreased *Epsilonproteobacteria*—increased *Deltaproteobacteria*—decreased
	γ-rays TBI Single dose 8 Gy		
Li Yiyi, 2020 [32] / Interventional	Mice C57BL/6J Male 6- to 8-week-old	16s rDNA / Fecal N = 2 1 week 6 weeks	Diversity/richness OTU number decreased Species number decreased Shannon diversity index—decreased Composition Bacteroidetes—decreased Firmicutes—decreased 1 week after, increased 6 weeks after Proteobacteria– increased significantly 1 week after, no alteration 6 weeks after Actinobacteria—decreased 1 week after, increased 6 weeks after Epsilonbacteraeota– decreased 1 and 6 weeks after *Bacteroides*, *Alistipes*, *Alloprevotella*, *Dubosiella*, *Rikenellaceae*, *Muribaculaceae*, *Enterococcus*, *Escherichia*, *-Shigella*, *Lachnospiraceae*—significant abundance changes in 1-week post-radiation as compared with unirradiated group. This effect is largely reversed in chronic phase of the disease *Eggerthellaceae*—significant abundance changes in 1-week post-radiation as compared with unirradiated group *Lactobacillus*—increased abundance in 6 weeks post-radiation *Akkermansia*—increased 6 weeks post-radiation
	X-rays Single dose 18 Gy 500 cGy/min for abdominal colorectal localized external radiation		
Raber J, 2020 [33] / Interventional	Mice C57BL/6 F1 4–6 months / N = 99	16S rRNA V4 region / Illumina Miseq / Fecal N = 1 2 months post-radiation	Diversity/richness Gut microbiome biodiversity (i.e., alpha-diversity), whether quantified as community richness or by using measures that combine community richness and evenness (e.g., Shannon entropy, Simpson’s diversity index), did not significantly vary as a function of radiation exposure or radiation dose. Composition The overall composition of the gut microbiome was significantly but weakly associated with radiation dose Many of the ASVs that differentially associate with radiation are members of the *Turicibacter* genus.
	Protons, ^4^He, ^16^O, ^28^Si, ^48^Ti and ^56^Fe ions		
Tong JY, 2022 [34] / Interventional	Mice C57BL/6J Female 3 weeks / N = 24 Controls = 6	16S rRNA V4 region / Illumina MiSeq / Fecal	Diversity/richness Shannon and Simpson indices—unchanged Chao and Ace indices—unchanged Composition Firmicutes, Bacteroidetes, Patescibacteria and Deferribacteres were the four dominant phyla At the phylum level—unchanged Firmicutes/Bacteroidetes *ratio* in the test group was higher than controls; the differences were not confirmed by statistics. *Moraxellaceae* and *Enterobacteriaceae*—significantly decreased *Lachnospiraceae*—significantly higher *uncultured_bacterium__Acinetobacter*, *uncultured_bacterium_o_*, *Mollicutes_RF39*, *uncultured_bacterium__Citrobacter* and *uncultured_bacterium_g_Lactococcus*—decreased
	X-rays TBI 5 groups: Test; 0.05, 0.10, 0.15 and 0.20 Gy		
Cheema AK, 2021 [35] / Interventional	Mice CD2F1 Male 6/7 weeks / N = 16/group	16S rRNA V3/V4 region / Illumina MiSeq SILVA / Fecal N = 5 7 and 1 days before irradiation and 3, 14 and 30 post-irradiation	Composition Firmicutes/Bacteroidetes *ratio*—altered∙ *Lactobacillus*—decreased∙ *Bacteroides* and *Alloprevotella*—increased
	γ-rays Single dose 9.2 Gy		

**Table 3:** Summary of study characteristics, demographics, radiation type, sample collection and analysis, and main findings of the eligible studies in animals (except mice).

Animals	Author, Year / Interventional	Participants / N Irradiated	Microbiome Assessment Method / Type of Sample / Number of Samples	Main Findings
		Type of Radiation		
Rats	Rentea RM, 2016 [36] / Interventional	Rats WAG/RijCmer Male 5 weeks / N = 15 5—Nonirradiated; 5—irradiated; 5—intestinal alkaline phosphatase (RT + IAP)	16s rRNA / Real-time PCR / Fecal N = 2 D0 and 4 days after irraiation	Composition Bacteroidetes—unaltered Firmicutes—slightly decreased Proteobacteria—greatly increased (100,000 xs)
		X-rays 13 Gy—single dose / Intestinal lower hemibody radiation		
	Lam V, 2012 [37] / Interventional	Rats WAG/RijCmcr (Wistar) Male 5 weeks / N = 10 (n = 5/group)	qPCR and 16S rRNA / Second Genome Inc. G3 PhyloChipe 16S rRNA microarray-based assay / Fecal N = 4 D0 and days 4, 11, and 21 post-irradiation	Composition Proteobacteria increased almost 1000-fold 4 days after 10 Gy and then returned to control values. 18 Gy prolonged increase over 5 days compared to over 3 days observed after 10 Gy Bacteroidetes—less affected *Cyanobacteria* OTU 31,902 increased *Clostridia*—less affected *Clostridia* OTU 39,153 decreased OTU 42,924 unchanged *Bacteroidales*—increased *Lactobacillaceae* and *Streptococcaceae*—increased *Peptostreptococcaceae*—unchanged *Clostridiaceae*—unchanged abundance but 47 separate OTUs decreased
		X-rays TBI Single dose 10.0 Gy Multiple-fraction 18.0 Gy		
Wild rodent: Bank Vole *Myodes glareolus*	Lavrinienko A, 2018 [38] / Observational	Wild rodent: Bank Vole Myodes glareolus / N = 137	16S rRNA V4 / Illumina MiSeq platform at BGI / Fecal	Diversity/richness Neither community richness nor evenness differed significantly (*p* > 0.05) between samples grouped by study area Significant differences in beta diversity Composition Radiation was identified as a significant predictor of the abundance of Bacteroidetes, Firmicutes and Proteobacteria (*p* = 0.001) *ratio* of Firmicutes to Bacteroidetes decreased Some members of the *Desulfovibrionaceae* can tolerate high radiation levels (CH) and have a potential for bioremediation of radionuclides
		3 study areas of environmental radiation: (1) high (CH) and (2) low (CL and KL)		
	Lavrinienko, 2020 [39] / Observational	Wild rodent: Bank Vole Myodes glareolus / 28 individuals provided fecal (CL1 n = 3, CL2 n = 13; CH1 n = 8, CH2 n = 4). (84–43 Recapture)	16s rRNA V4 / Illumina MiSeq platform at BGI / Fecal N = 1	Diversity/richness alpha diversity (number of ASVs, Shannon Index) unchanged. Composition Enrichment of members of the S24-7 family (Bacteroidetes) in samples from CL and an increase in ASVs assigned to Ruminococcaceae, Lachnospiraceae (Firmicutes) and Desulfovibrionaceae families in CH samples. Second capture CL: S24-7 family (>10% reduction in relative abundance) decreased *Ruminococcaceae* and *Lachnospiraceae* (*p* < 0.05)—increased
		Ambient radiation Chernobyl High Radiation (CH) and Chernobyl Low radiation (CL)		
Göttingen minipigs and Chinese rhesus macaques	Carbonero F, 2018 [40] / Interventional	Göttingen Minipigs and Chinese rhesus Macaques 8 Minipigs 8 Macaques	16s rRNA / Illumina MiSeq / Fecal N = 2 -2/3 days before -3 days after	Minipigs Diversity/richness Shannon index—decreased Composition *Clostridiales*—increased *Bacteroides* and *Paraprevotella*—decreased *Blautia*, *Oscillibacter*, *Streptococcus* and *Lactobacillus*—increased *Roseburia*, *Ruminococcus* and *unclassified Lachnospiraceae*—Significant decreased Macaques Diversity/richness No significant effect diversity indices (taxa number and Shannon index) Composition Verrucomicrobia increased unclassified Lachnospiraceae and Veillonellaceae—increased Both *Helicobacter* increased *Treponema*, *Elusimicrobium* increased
		6 MV linear accelerator (LINAC) 80 ± 2.5 Gy/min 1.8 Gy Minipigs 6.8 Gy Macaques		
	Carbonero F, 2018 [41] / Interventional	Göttingen minipigs Chinese rhesus macaques / N = 74 male Chinese rhesus macaques 50 Minipigs	16s rRNA / Illumina MiSeq QIAGEN / Fecal / Minipigs: collected on days 0 and 3 Macaque fecal samples were collected 24 h before irradiation, between 1–3 h postirradiation and on days 3 and 14 postirradiation	Macaques Diversity/richness Overall diversity was not significantly affected Number of taxa observed decreased numerically (66 to 63) Composition Firmicutes decreased Spirochaetes increased Actinobacteria decreased Proteobacteria and Bacteroidetes—increased *Helicobacter* and *Treponema*—decreased/only higher radiation levels—immediate increase. *Betaproteobacteria* members, Desulfovibrio and Bilophila—decreased *Streptococcus* and *Prevotella*—decreased *Bacteroides* and *Parabacteroides*—increased *Paraprevotella* and *Clostridium clusters IV* and *XIVa*—increased *Clostridium* increased *Clostridium XIVa* Significant positive correlations *Blautia* and *Lactobacillus* increased Actinobacteria major genera—decrease (*Collinsella* and *Slackia*) *Collinsella* higher radiation levels were characterized by lower numbers *Slackia* higher radiation levels—lower numbers Minipigs Richness Number of genera—increased Composition Firmicutes and Verrumicrobia increased Bacteroidetes and Proteobacteria decreased *Bacteroides*, *Clostridium*, *Roseburia*—decreased *Streptococcus* increased *Oscillibacter* increased/correlated negatively with radiation intensity *Blautia* increased Elusimicrobium All radiation levels led to significant decreases/were found to correlate negatively with radiation intensity until 2.1 Gy. *Prevotella*, *Faecalibacterium*, *Bifidobacterium* decreased *Clostridium cluster IV*, *XIVa* and *XIVb*: High radiation levels (1.95–2.25 Gy) led to increases *Olsenella* and *Alistipes*—increased *Butyricimonas* and *Collinsella*—decreased *Ruminococcus* and *Clostridium XIVa*—significant positive correlations *Lactobacillus* correlate negatively
		Macaques 5.9 Gy (n = 12); 6.3 Gy (n = 14); 6.8 Gy (n = 16); 7.2 Gy (n = 16); and 7.7 Gy (n = 16) Minipigs 1.65 Gy (n = 9); 1.80 Gy (n = 10); 1.95 Gy (n = 11); 2.10 Gy (n = 13); and 2.25 Gy (n = 7)		
Chinese rhesus macaques, Macaca mulatta	Kalkeri R, 2021 [42] / Interventional	Chinese rhesus macaques, Macaca Mulatta / N = 19	Fecal samples / N = 3 1 day prior and 1 and 4 days after exposure	Diversity/richness Alpha Diversity (Shannon Diversity Index) revealed no major difference between pre- and post-irradiation, Beta diversity analysis showed significant differences in the microbiome after irradiation (day + 4) compared to baseline (pre-irradiation) Composition Firmicutes/Bacteriodetes *ratio*—decreased *Actinobacillus*, *Bacteroides*, *Prevotella* (*Paraprevotellaceae* family) and *Veillonella*—significantly increased *Acinetobacter* and *Aerococcus*—decreased
		Gamma-rays 7.4 Gy		
Flies	Cai Z, 2018 [43] / Interventional	Flies Males Bactrocera dorsalis 3000 pupae irradiated 15 guts irradiated 15 guts control	16s rRNA V4 / Illumina MiSeq QIIME v1.8 / Gut / Irradiation 48h before eclosion Day1 Day7 Day14 Post eclosion	Diversity/richness Diversity significant increase at 1 DPE (ACE, Chao1, Shannon indexes). At 7 DPE, the ACE, Chao1 and Shannon indexes increased Chao1 index—significant difference between irradiated and control flies, at 7 DPE. Richness increased Total bacteria decreased by 40% at 1 DPE. No significant differences at 7 or 14 DPE Composition *Enterobacteriaceae* decreased 54% at 1 DPE, 52% at 7 and 51% at 14 DPE *Bacillaceae*, *Clostridiaceae*, *Xanthomonadaceae*, *Sphingobacteriaceae*, *Aeromonadacea* and *Flavobacteriaceae* increased significantly
		100Gy gamma ray Gammacell 220 60Co With an activity of 9435 × 1015 Bq Central dose of 8Gy/min at the beginning of the test		
	Ben Ami, 2020 [44] / Interventional	Flies Vienna 8 Wild C capitata pupae / 150 bacterial colonies from non irradiated 150 colonies from 5-day-old irradiated flies and 100 colonies from field flies	16s rRNA / PCR-DGGE	Diversity Gut bacterial diversity, as expressed by the total number of bands appearing in the gel, is reduced at eclosion day in the irradiated gut compared with non-irradiated guts and to those of 5-day-old males (3.47 ± 0.22 bands per lane for the irradiated eclosion day gut compared with 5.3 ± 0.39 and 5.55 ± 0.62 bands per lane for the non-irradiated eclosion day gut and 5-day-old gut, respectively) Composition Non-irradiated vs. irradiated vs. irradiated mass 5 day-read *Klebsiella* sp.—18.67% vs. 4.0% vs. 23.0%. Is a dominant community among the total gut microbiota of the non-irradiated, 5-day-old irradiated flies and of wild flies (18.67, 23.0, and 31.0%, respectively); its prevalence in the gut of the irradiated flies on eclosion day is significantly lower (4.0%, t-test: t ¼ 2.0129, *p* < 0.05) *Enterobacter* sp.—21.33% vs. 37.33% vs. 23.0% *Citrobacter* sp.—9.3% vs. 4,6% vs. 4.0% *Bacillus* sp.—8.0% vs. 7.33% vs. 2.0% *Pseudomonas* sp.—20.67% vs. 27.33% vs. 16.0% *Ralstonia* sp.—10.0% vs. 8.67% *Providencia* sp.—12.0% vs. 4.0% vs. 22.0%
		Delta irradiation		
	Woruba DN [45] / Interventional	Flies Queensland fruit fly, Bactrocera Tryoni 54 = (3 × 18)	16S rRNA V3 and V4 regions QIIME / Intact gut dissections / N = 2 1 and 14 days after irradiation	Diversity/richness Diversity increased No changes in bacterial diversity and in relative abundance of OTU Bacterial load increased
		Delta irradiation		

No formal statistical analysis was undertaken due to the small number of retrieved eligible studies and the heterogeneity of the data and outcomes presented.

## 3. Results

### 3.1. Search Results

A total of 5224 citations were identified: 2852 through PubMed, 2914 through EMBASE and 87 through Cochrane library (Figure 1). After removing duplicates and adding two citations from reference lists, 3531 papers were screened for inclusion based on their titles and abstracts. A total of 3450 were excluded, and the full text of the remaining 82 studies was evaluated; a further 53 were then excluded (eleven were studies in humans, thirty did not report the effect of ionizing radiation in microbiota, five were literature revisions; four were commentaries; one was written in a language unreadable by the authors, and the authors were not able to access one article full text). The two reviewers found a final total of 29 studies eligible for review, with a perfect agreement between them (κ = 1).

### 3.2. Study Characteristics

Twenty-seven interventional studies and two observational studies were included. The analyzed studies were quite heterogeneous regarding population, study methodology, and outcomes. A summary of the characteristics of the studies is presented in Table 2 and Table 3.

#### 3.2.1. Animal Models

Most studies analyzed the gut microbiota from mice (15 used substrains of C57BL/6 [17,18,19,20,21,22,26,27,28,32,33,34], one used BALB/c [23], one used CD2F1, [35] and one other study did not specify the strain [30]), two studies used rats (WAG/RijC) [36,37], two used wild bank voles (*Myodes glareolus*) [38,39], three used Chinese rhesus macaques [40,41,42], two used Göttingen minipig, [40,41] and three analyzed the gut microbiota of flies [43,44,45].

Nineteen studies evaluated the shift of the gut microbiota of mice, and three studies evaluated rats. Given their small size, low maintenance costs, relatively stable embryonic cells and pliability for genetic manipulations and gene editing, mice are considered the preferable animal model to study human gene functions. However, rats are physiologically, morphologically and genetically closer to humans than mice, which makes rats ideal models for biomedical and clinical studies [46].

Three studies used minipigs and nonhuman primate models. These animals represent large pre-clinical models which have demonstrated physiologic, anatomic, proteomic and genomic similarities to humans [40,47,48]

#### 3.2.2. Radiation Exposure Characteristics

The type of radiation exposure varied throughout the studies. Most researchers evaluated the effect of ionizing radiation from artificial exposure to X-rays [18,19,24,25,32,36,37] or gamma rays [17,20,21,22,23,28,29,31,43,45] in either single or multiple doses, while one used delta radiation [44].

Most researchers used standard total body irradiation (TBI) models [17,19,20,26,30,31,37,42], while some used total abdominal irradiation (TAI) [17,25,29] or localized internal rectal irradiation [22] models to study the effects of irradiation on the gut microbiome. The gamma and X-rays doses ranged from 10 to 18 Gy in TAI studies and 0.1 to 12.0 Gy in TBI. Total abdominal irradiation and total body irradiation ranged from 0.1 Gy to 19.0 Gy in mice studies, from 5.9 Gy to 7.7 Gy in macaques and from 1.8 Gy to 2.25 Gy in minipigs.

Space travel is associated with continuous low-dose-rate exposure to radiation that might affect the gut microbiota. Two studies evaluated the effect of space-type radiation, exposing mice to high-energy transfer protons and ions [26,49].

The Chornobyl disaster provides a unique environmental opportunity to explore the impacts of chronic exposure to low-dose radioactive contaminants. Lavrinienko et al. conducted two studies to evaluate the gut microbiota of wild bank voles (*Myodes glareolus)* exposed to natural environmental radiation in areas of the Chornobyl exclusion zone that differed in the level of radionuclide contamination. *Myodes glareolus* is a small rodent that is an important mammalian wild model of the biological effects of exposure to ionizing radiation because it combines ecological relevance with laboratory tractability [38,39].

Finally, ionizing irradiation is often used to sterilize insects. However, it may have negative side effects on male insects’ fitness, resulting in reduced competitiveness. Three studies analyzed the shifts of the gut microbiota of flies, exposing them to high doses of gamma-rays (from 65 Gy to 100 Gy) [43,44,45].

#### 3.2.3. Sampling and Microbiota Analysis

Most studies performed in mammals included in this review characterized the gut microbiota through fecal samples collected from the cages [17,18,21,35,42] or removed directly from the terminal ileum, cecum or rectum [28,29,30]. Differently, Johnson et al. analyzed tissue samples from the irradiated small intestine [24]. The studies performed in flies analyzed intact gut dissections [43,44,45].

Fecal samples are considered the most convenient collection method. They are easier to sample frequently, are non-invasive and have long been used for the analysis of the distal gut microbiota. Fecal samples have the disadvantages that they might contain inactive bacteria, bacteria from other gastrointestinal tract compartments, and less controlled sampling variables when compared to biopsy [50].

The number of obtained samples was very heterogeneous between studies varying from one to eight samples at different time points from the same animal. Some studies only collected one sample and compared it to controls, while other studies compared before and after exposure to ionizing radiation.

The sampling collection times within the studies were also very heterogeneous, ranging from after exposure to up to 10 months post-exposure.

Furthermore, the methodology used to study microbiota varied in the different studies.

Most studies chose 16S rRNA-based sequencing [17,20,21,27], whereas a few used qPCR [18,30]. In one study, an older method was used based on bacterial culture colony-forming units [24].

Richness, assessed by the number of OTUs/species, and diversity (alpha diversity and beta diversity) were parameters evaluated in most of the reviewed studies.

Most studies calculated alpha diversity through the Chao1 index, Shannon’s index and Simpson’s index. For beta diversity Bray–Curtis dissimilarity, Un-weighted UniFrac and Weighted UniFrac were used.

### 3.3. Quality Assessment

During the quality assessment, the reviewers verified that none of the selected studies reported methods of sequence generation or concealed allocation. Regarding the same baseline characteristics, most studies chose animals of the same ages and sex, but few specifically mentioned the weight of the different animals. One of the assessed studies did not specify the used animal’s baseline characteristics.

Regarding random housing, the reviewers considered that it is unlikely that the outcome measurement was influenced by not randomly housing the animals as they all followed the ethical rules for animal studies. None of the studies reported the blinding of the caregivers/investigators. The reviewers considered that although the outcome assessor was not blinded, the outcome, due to its characteristics, is not likely to be influenced by a lack of blinding.

Regarding attrition bias, most studies are not clear regarding how many animals were considered initially, so it was impossible to determine if all the considered animals were analyzed.

Finally, the reviewers considered that there was a low reporting bias.

Detailed information regarding t the quality assessment of the included studies are presented in Table S1 (Supplementary File).

### 3.4. Findings

The analyzed studies suggest that ionizing radiation causes significant changes in the composition, diversity, and richness of the gut microbiota. The key findings of the studies are organized in Table 4.

#### 3.4.1. Diversity and Richness Analysis

Overall, studies reported that the diversity of the gut microbiota was altered by ionizing radiation. The α diversity, measured by Shannon, Simpson, ACE and/or Chao1 indexes, decreased in most studies that evaluated diversity (13 in 21 studies) [17,19,21,23,25,26,29,32,40,44], and five studies described increases in α diversity.

β diversity was evaluated in 6 studies, and 5 found significant differences [17,23,26,38,39,42].

Fourteen studies described the effect of IR on richness and most demonstrated that ionizing radiation decreases richness, as measured by the number of OTUs/taxa number and richness/Chao1 index [21,23,29,31,32,43]. Two studies reported that the richness and diversity remained unchanged [40,45]. The studies that reported the increase in richness were the studies with flies [43,45].

The study that used the cultured-based method could not assess these parameters, which was expectable [24].

#### 3.4.2. Gut Microbial Composition

Almost all studies reported changes in the microbiota composition after exposure to IR, suggesting that localized irradiation dramatically altered gut microbial composition [17,20,22,25,30,32]. However, the methodology of results reporting was widely variable among them. Some only analyzed alterations at the phylum or genus level, while only three studies analyzed species level [19,26,31]. The qPCR and culture-based studies had limited results of the specific taxa analyzed [18,24,30].

At the phylum level, one of the most consistent findings was the increase of the Proteobacteria following radiation exposure (90% of the studies that reported changes in Proteobacteria relative abundance) [23,25,27,28,29,32,36,37,41]. The most significant increases were found in Lu L et al.’s research (rise of 20%) [25] and in Zhao Z et al.’s (raised from 7.4 to 22.0%) [29]. In Lam V et al.’s research, the abundance increased almost 1000-fold 4 days after 10 Gy of total-body irradiation but then returned to control values [37]. Additionally, the family *Desulfovibrionaceae*, from the Proteobacteria phylum, showed a significant increase in two studies [17,39].

Contrarily, the relative abundance of Firmicutes decreased in most studies [19,21,22,25,30,36,40,42]. In Li Yiyi et al. study, Firmicutes decreased one week after but increased six weeks after [32]. In another study, the abundance in the large intestine tended to be lower but increased the amount in the small intestine by approximately 18 percentage points [27].

The relative abundance of the phylum Bacteroidetes decreased in four studies, [23,28,32,40] increased in one study [27] and was not significantly affected in another two [36,37].

The *ratio* Firmicutes to Bacteroidetes decreased in four studies [21,28,38,42] and increased in one study but without significance [34].

The abundance of Verrucomicrobia increased in 75% of the studies [19,27,30,40,41]. Contrarily, in Zhao Z et al.’s research, the abundance decreased from 2.9 to 0.0006% [29].

Finally, the abundance of Actinobacteria decreased in two studies [27,40] and increased in one study [25]. In Li Y et al. research, the abundance decreased one week after exposure and increased six weeks after [32].

At the genus level, the findings were less consistent. *Lactobacillus* decreased in most studies [18,21,24,28,35] and increased in two studies [27,32]. Four studies showed a decrease in *Bacteroides* [23,27,30,41] and an increase in two [28,41].

The abundance of *Akkermansia* increased in three studies [27,30,32]. *Alistipes* increased in four studies [28,30,41] and decreased in one study [25]. Interestingly, in Kim Y et al.’s research, there was an increase of the genus in the large intestine and a decrease in the small intestine [27].

*Bifidobacterium* decreased after exposure in three studies [18,26,41]. In the study performed by Yamanouchi et al., a mixed response was found. In the 2 Gy–irradiated group *Bifidobacterium* presented a decreasing trend from 6 h after irradiation, which continued until 72 h. But the 4 Gy–irradiated group presented an increase of ~10 times after 48 h, reaching 28 times after 72 h [18].

## 4. Discussion

Animal models are a powerful tool for studying the underlying mechanisms of gut-microbiota-associated diseases and might help to understand the shifts after exposure to ionizing radiation.

This review provides a detailed overview of the pre-clinical studies describing the effect of ionizing radiation on the gut microbiota diversity, richness, and composition of animals. Most studies consist of controlled laboratory assays on small animals, especially on mice.

The mouse and human microbiota are quite similar at the phylum level, with Firmicutes and Bacteroidetes being the most frequent. However, most of the gut composition is unique. At least 85% of the sequences representing genera in mice are not detected in humans [13,51,52], and some important genera that are frequent in humans are not detected in some laboratory mice, such as *Faecalibacterium* [53]. Nevertheless, animal models provide some relevant insights into the direct effect of radiation, namely for identifying the most radiosensitive bacteria. 

These models allow a detailed study of the inflammatory process and of the complex interactions occurring between the host and the intestinal microbiota [51]. Other limitations of animal models include differences in enzyme activity, concentrations of putrefactive products, and immunological activation by the feces content [51,54].

Overall, the analyzed animal experiments confirm that ionizing radiation causes significant changes in gut microbiota composition, diversity and richness.

Interestingly, despite multiple different outcome measures, some concordant results emerged.

Most studies showed a decrease in diversity (especially alpha diversity), the most common finding in dysbiosis, [55] with multiple studies describing lower diversity as being associated with various diseases such as inflammatory bowel disease, [56,57] type 1 diabetes, [58] and obesity [59].

Concerning composition, at the phylum level, the gut microbiota of the analyzed animals was mainly composed of Firmicutes, Bacteroidetes, Proteobacteria, Actinobacteria and Verrucomicrobia [60]. Although different results were found throughout the studies, one of the most consistent findings following exposure to ionizing radiation, also observed in human studies [14], was the increased relative abundance of Proteobacteria [23,25,27,28,29,36,37]. The enrichment of Proteobacteria is considered a sign of dysbiosis and has been associated with multiple pathologies, including inflammation [28,29].

The decrease in the relative abundance of both Bacteroidetes [23,28,40] and Firmicutes [19,22,25,30,36,40] was another frequent finding. Like in human studies, four experiments described a decrease in the Firmicutes/Bacteroidetes ratio [21,28,38,42]. The association between these two dominant phyla has been related to several pathological conditions, including obesity [61]. However, the F/B ratio is considered a controversial measure since it only focuses on a high-level taxonomic rank. More recent studies that also analyzed other taxa levels (genus, species, or strain) suggest that the complexity of disease modulation by gut microbiome is much more complex than only an imbalance of these two phyla [62].

Other frequent findings were the increase of the Verrucomicrobia phylum [19,27,30,40,41] of its genus *Akkermansia* and of the specie *Akkermansia muciniphila* (*A. muciniphila*) [26,27,30,32]. *Akkermansia* is known to have an important value in improving host metabolic functions and immune responses [63], and several studies reported a reduction in the abundance of *A. muciniphila* in various human diseases, including inflammatory bowel disease, autism, atopy and obesity [64,65].

At the genus level, despite conflicting findings (showing either an increase or a decrease in each genus), the most consistent finding was the decrease in the relative abundance of the genera *Bifidobacterium* [18,26,40] and *Lactobacillus* [18,21,24,28,66] well known for their probiotic effects and shown to be beneficial for the host, being used in clinical practice for gastrointestinal diseases [18,67,68]. *Lactobacillus* has also been linked to an increase in survival rates after IR exposure [69].

The increase in the genus *Alistipes* was another consistent finding [27,28,30,41]. *Alistipes* have been seen to have a protective role in multiple diseases, including colitis, autism spectrum disorder and fibrotic liver disorders, but have also been found to contribute to disease [70,71,72].

Regarding other taxa levels, such as order, family, genus, species, or strain, multiple significant findings were found but were dispersed and are summarized in Table 4.

Although gamma rays are typically more energetic than X-rays, so they have a more ionizing effect compared to X-rays, we did not find differences in the effect on gut microbiota.

Concerning the radiation doses, interestingly, Casero et al. found a higher sensitivity of the gut microbiota to lower doses—0.1 and 0.25 Gy as compared to the highest dose—1 Gy, suggesting that at higher doses, DNA repair mechanisms were fully in effect and resulted in a seeming reduction in radiosensitivity [26]. It should be taken into account that some microorganisms are resistant to higher levels of ionizing radiation. Bacterial survival and adaptation to stressors include a complex regulation network, including post-transcriptional regulators, such as small RNAs, which may enhance bacterial resistance to ionizing radiation when adequately combined [73,74].

Ionizing radiation can have significant molecular effects on the gut microbiota, leading to microbial composition, metabolism, and function alterations. The possible molecular effect of ionizing radiation on the gut microbiota is the induction of oxidative stress by generating reactive oxygen and nitrogen species (ROS/RNS) that can damage cellular components and impair the cellular functions of the bacteria. ROS/RNS can also alter the gut microbiota by changing the redox state of the intestinal environment and affecting microbial growth, survival, and metabolism. Another possible major effect of IR on the gut microbiota is the modulation of microbial gene expression that can lead to alterations in microbial metabolism and function [4,5,6,7]. IR can also modify the gut microbiota’s composition by promoting certain microbial species’ growth and suppressing others.

When intestinal inflammation occurs after IR exposure, the oxygen levels are increased. This event leads to an increase in facultative aerobes such as Proteobacteria. It has also been described that oxidative stress actively stimulates the enrichment of Proteobacteria [35]. Unlike obligate anaerobic members of the gut microbiota, the facultative anaerobic can use nitrate, S-oxides and N-oxides as terminal electron acceptors for anaerobic respiration. In the present review, we found that after IR exposure, the relative abundance of the two major groups of anaerobes, Firmicutes and Bacteroidetes, decreased, and the relative abundance of Proteobacteria, an important group of facultative anaerobic bacteria, increased [35,75,76]. 

The increase in *A. muciniphila* can be explained by two factors. First, this bacterium can tolerate a small amount of oxygen; additionally, it belongs to the mucin-degrading bacterial family and can generate energy by decomposing mucin secreted by the gut mucosa. *A*. *muciniphila* uses mucin as its sole carbon and nitrogen source and produces enzymes that destroy mucin. Due to these facts, when more mucin is present, *A. muciniphila* has a competitive advantage over other bacteria and increases its relative levels on the local microbiota [64,77].

There were limitations in this review. The primary limitation is that the number of irradiated animals varied greatly across the studies and that most trials had small sample sizes (most studies that exposed mammals to radiation included less than ten animals), [17,18,27] which may condition the study results and their interpretation. The studies with the higher number of animals were those which included flies [43,44,45] and those that analyzed wild rodents exposed to environmental radiation [38,39].

In addition to the inclusion of different types of animals, there were also sex and age differences. Interestingly, one study that analyzed both female and male mice found significant outcome differences between the sexes [20]. Most studies only analyzed males or females; only one other study included both female and male mice and did not refer to differences in the results [17]. More studies including animals of both sexes and addressing possible differences in gut microbiota response to irradiation would be important.

Furthermore, the type, dosage, and duration of radiation exposure varied. Most studies analyzed the effect of acute artificial exposure to low-dose gamma or X-rays. Two studies analyzed acute exposure to high-energy space-type radiation [26,33], and two others analyzed the effect of chronic exposure in contaminated areas near Chornobyl [38,39]. It is known that radiation effects depend on the dose, dose rate, dose fractionation, irradiated volume and type of radiation. The interpretation of results from the different studies should take into consideration the type and characteristics of radiation exposure.

Another factor that should be considered is the method used for microbiota characterization. One study used a cultured-based method, and few used qPCR and primers. The latter has the disadvantage of limiting the information to the selected genera [18,24,30]. Most of the remaining studies chose 16S rRNA sequencing to study gut microbiota’s taxonomic distribution and diversity. In fact, 16S rRNA is a cost-effective semi-quantitative method [2]. Even though it is the most commonly utilized method, 16S rRNA presents some disadvantages. For instance, the identification accuracy depends on the size of the reference database, and the resolution power is only at the species level. However, most of the included studies only analyzed genus levels [78].

Methodologies such as metagenomics, metatranscriptomics, metaproteomics and metabolomics can be used to study functional gut microbiota. Shotgun metagenomics, a quantitative method that provides a large amount of functional information, allows identification at the strain level (low-level taxonomic rank describing genetic variants or species subtypes). However, it is costly and not used frequently in these studies [2].

Most studies used fecal samples. However, despite being the most common sampling method used, it may only partially represent the structure of the whole gut microbiota.

Finally, the time points of feces collection after exposure also varied, and several studies did not evaluate long-term effects [18,25,36]. Most of the studies that had long-term evaluations reported changes immediately after exposure to ionizing radiation but found they were not permanent [18,19,21,23,26,30,37].

## 5. Conclusions

Animal models allow the investigation of the effect of ionizing radiation without some of the confounding factors and limitations that exist in human studies. The studies included herein demonstrated that dysbiosis occurs after exposure to ionizing radiation. All studies demonstrated shifts in composition, richness, or diversity, highlighting the importance of considering the effects of ionizing radiation exposure on the gut microbiota.

Overall, several limitations were identified as the population, methodology and the reporting of outcomes were highly variable throughout the included studies, which renders comparisons of the multiple findings rather difficult, with multiple conflicting outcome measures.

Despite the mentioned limitations, consistent and convincing evidence was found: diversity and richness are reduced after ionizing radiation exposure. Some consistent findings were also found regarding composition. At the phylum level, Firmicutes and Bacteroidetes’ relative abundance decreased, while Proteobacteria’ and Verrucomicrobia’ increased. At the genus level, *Alistipes* and *Akkermancia* increased in most studies, while *Lactobacillus* decreased. These findings should be further explored and considered, especially when considering the side effects of medical treatments and further embracing prophylactic/therapeutic attitudes.

Notably, significant coincident findings between human and animal studies were found, namely the decrease of alfa diversity and richness; the decrease of the ratio Firmicutes/Bacteroidetes; the decrease of Firmicutes; the increase of Proteobacteria. At the genus level, in most studies, the decrease in *Lactobacillus* [14].

Importantly, we did not find significant contradictory results. In animal studies, we have relevant results in Verrucomicrobia, *Alistipes* and *Akkermansia*, but in human studies, these groups of bacteria were not evaluated.

More extensive, better-designed studies and longer time horizons are needed to better understand and characterize the process and the influence of IR on the gut microbiome.

## Figures and Tables

**Table 1:** Literature search algorithm—PubMed; EMBASE (via OVID); and Cochrane Library.

Database	Search Number	Search Terms
PubMed	Search #1	“microbiota” OR “gastrointestinal microbiome” OR “microbiome” OR “16s rRNA”
	Search #2	“radiation” OR “radiotherapy”
	Search #3	Search #1 AND Search #2
EMBASE (via OVID)	Search #1	“microbiota” OR “gastrointestinal microbiome” OR “microbiome” OR “16s rRNA” OR “microflora”
	Search #2	“radiation” OR “radiotherapy”
	Search #3	English OR Spanish OR Portuguese
	Search #4	Search #1 AND Search #2 AND Search #3
Cochrane Library	Search #1	“microbiota” OR “gastrointestinal microbiome” OR “microbiome” OR “16s rRNA” OR “microflora”
	Search #2	“radiation” OR “radiotherapy”
	Search #3	Search #1 AND Search #2

**Table 4:** Key findings from selected studies.

Key Findings from the Studies	
Diversity	
Altered in males but not in females (Cui M, 2017) [20] Decreased (Sittipo P, 2020) [21] Decreased at eclosion day in the irradiated gut males (Ben Ami, 2020) [44] Decreased (Casero D, 2017) [26] Significant increase at 1 DPE (Cai Z, 2018) [43] Increased (Woruba DN) [45] Macaques: not significantly affected (Carbonero F, 2018) [41]	
ACE index	Unaltered (Li Y, 2020) [17] Significantly higher (Cai Z, 2018) [43]
α diversity	
Decreased *p* < 0.05 (Lu L, 2019) [25] Decreased slightly on day 6 (Li Y, 2020) [17] Did not cause significant changes 12 Gy dose (Goudarzi M, 2016) [19] No significant differences (Wang W, 2020) [30] No significant difference (Tong, 2022) [34] Unchanged (Raber J, 2020) [33] Marked increase 30 days (Casero D, 2017) [26]	
Shannon index	Decreased, no statical differences (*p* = 0.055) (Lu L, 2019) [25] Decreased. Recovers 30 days after. (Goudarzi M, 2016) [19] Unaltered (Wang M, 2020) [28] Decreased significantly; (*p* < 0.05) (Liu X, 2019) [23] Decreased *p* = 0.97 (Sittipo P, 2020) [21] Decreased significantly; *p* = 0.03 (Zhao Y, 2019) [31] Remarkably decreased *p* < 0.0001 (Li Yiyi, 2020) [32] Unaltered (Tong, 2022) [34] Macaques: no significant effect/Minipigs: decreased (Carbonero F, 2018) [40] Increased (60% significantly) (Cai Z, 2018) [43] No major difference between pre- and post-irradiation (Kalkeri, 2021) [42] No significant difference (Lavrinienko, 2020) [39] Increased in small intestine and no significant differences in large intestine (Kim YS, 2015) [27]
Simpson diversity index	Significantly greater *p* = 0.0440 (Zhao Z, 2020) [29] No significant difference (Wang M, 2020) [28] Increased significantly (Liu X, 2019) [23] Unaltered (Tong, 2022) [34]
Chao1 index	Lower, no statically different *p* = 0.069 (Lu L, 2019) [25] Significant lower *p* = 0.0120 (Zhao Z, 2020) [29] Decreased on day 3. Recovered 30 days after. (Goudarzi M, 2016) [19] No significant difference (Wang M, 2020) [28] Decreased *p* = 0.015 (Sittipo P, 2020) [21] Unaltered (Li Y, 2020) [17] Unaltered 10 months after *p* = 0.64 (Zhao Y, 2019) [31] Unaltered (Tong, 2022) [34] Increased (Cai Z, 2018) [43] Increased in small intestine and no significant differences in large intestine (Kim YS, 2015) [27]
Beta diversity	
Changed (Li Y, 2020) [17] Significantly different in the LT10 (Liu X, 2019) [23] Differences (Kalkeri, 2021) [42] Significant differences (*p* = 0.001) (Lavrinienko A, 2018) [38] Significant difference *p* < 0.001 (Casero D, 2017) [26] Unchanged *p* = 0.12 (Zhao Y, 2019) [31]	
Richness	
Number of OTUs/Taxa number	Significantly lower (Zhao Z, 2020) [29] Decreased significantly as LDR exposure time increased. However, no difference was found among groups LT1, LT5, and LT10 (*p* < 0.05). (Liu X, 2019) [23] Diversity decreased—OTUs estimated by richness analysis (*p* = 0.009) (Sittipo P, 2020) [21] Different OTUs after (Goudarzi M, 2016) [19] Remarkably decreased (Li Y, 2020) [32] Decreased species number significantly (*p* < 0.05). (Liu X, 2019) [23] Increased bacterial load (Woruba DN, 2019) [45] Decreased by 40% at 1 DPE (Cai Z, 2018) [43] No significant differences (*p* > 0.05) (Lavrinienko A, 2018) [38] Higher in small intestine and no significant differences in large intestine (Kim YS, 2015) [27]
Altered composition/Dysbiosis	
Marked dysbiosis (Lu L, 2019) [25] Intestinal bacterial flora substantially shifted (Li Y, 2020) [17] Altered composition of enteric bacteria in males but not in females (Cui M, 2017) [20] Significant shift in post-radiation gut microbial composition (Gerassy-Vainberg, 2018) [22] Not changed significantly 7 days after, with obvious changes 30 days after (Wang W, 2020) [30] Significant shift in microbial composition (Li Yiyi, 2020) [32] Composition associated with radiation dose (*p* = 0.0002) (Raber J, 2020) [33]	
Anaerobic counts	
	Significant decreases 2 and 6 h *p* <0.05 compared to 24 h. No significant differences 24 h after (Johnson, 2004) [24]
Aerobic counts	
	Significantly decreased; *p* <0.05. Compared to the 24 h levels, significant decreases at 2 h *p* < 0.05 (Johnson, 2004) [24]
Phylum	
*Ratio* Firmicutes/Bacteroidetes	Decreased (Sittipo P, 2020) [21] Decreased (Wang M, 2020) [28] Decreased (Lavrinienko A, 2018) [38] Increased, without significance (Tong, 2022) [34] Altered (Cheema, 2021) [35] Decreased (Kalkeri, 2021) [42]
Actinobacteria	Smaller increase (Lu L, 2019) [25] Decreased in large intestine (Kim YS, 2015) [27] Decreased 1 week after, increased 6 weeks after (Li Yiyi, 2020) [32] Decreased (Carbonero F, 2018) [41]
Bacteroidetes	Increased in the large intestine by 4 percentage points (Kim YS, 2015) [27] Decreased (Wang M, 2020) [28] Decreased in minipigs (Carbonero F, 2018) [41] Significantly decreased in a time-dependent manner (Liu X, 2019) [23] Decreased (Li Yiyi, 2020) [32] Unchanged (Rentea RM, 2016) [36] Less affected (Lam Vy, 2012) [37]
Epsilonbacteraeota	Decreased 1 and 6 weeks after (Li Yiyi, 2020) [32]
Firmicutes	Decreased at D1; recovered at later (D3 and D10) (Sittipo P, 2020) [21] Decreased (Lu L, 2019) [25] Decreased (Goudarzi M, 2016) [19] Decreased (Rentea RM, 2016) [36] Decreased—large intestine. Increased—small intestine (Kim YS, 2015) [27] Significantly decreased (*p* < 0.01). (Gerassy-Vainberg, 2018) [22] Decreased 30 day after (Wang W, 2020) [30] Decreased 1 week after, increased 6 weeks after (Li Yiyi, 2020) [32] Decrease in Macaques and Increased minipigs (Carbonero F, 2018) [41]
Proteobacteria	Increased (Lu L, 2019) [25] Significantly increased (Zhao Z, 2020) [29] Increased (Kim YS, 2015) [27] Increased (Wang M, 2020) [28] Increased (Liu X, 2019) [23] Increased (Rentea RM, 2016) [36] Significant change in abundance. (Gerassy-Vainberg, 2018) [22] Increased 1 week after, but no alteration 6 weeks after (Li Yiyi, 2020) [32] Increased 4 days after, then returned to control values. (Lam Vy, 2012) [37] Macaques—Increases/Minipigs—decreases (Carbonero F, 2018) [41]
Verrucomicrobia	Decreased (Zhao Z, 2020) [29] Increased. Recovered by day 30 (Goudarzi M, 2016) [19] Identified in the irradiated samples but not in the control samples. (Kim YS, 2015) [27] Akkermansia spp. (*p* < 0.01)—significant change. (Gerassy-Vainberg, 2018) [22] Increased (Wang W, 2020) [30] Increased minipigs and macaques (Carbonero F, 2018) [40] Increased minipigs (Carbonero F, 2018) [41] Increased (Casero D, 2017) [26]
Spirochaetes	Increases (Carbonero F, 2018) [41]
Class	
* Clostridia *	Increased (Liu X, 2019) [23] Decreased (Zhao Y, 2019) [31] Less affected (Lam Vy, 2012) [37]
*Bacteroida*	Significantly decreased in LT10 (Liu X, 2019) [23] Increased (Zhao Y, 2019) [31]
* Betaproteobacteria *	Increased (Zhao Y, 2019) [31]
* Unidentified_Saccharibacteria *	Decreased (Zhao Y, 2019) [31]
* Epsilonproteobacteria *	Increased (Zhao Y, 2019) [31]
* Deltaproteobacteria *	Decreased (Zhao Y, 2019) [31]
* Erysipelotrichia *	Increased (Zhao Y, 2019) [31]
Order	
* Clostridiales *	Increased in the LT10 group (Liu X, 2019) [23] Increased (Carbonero F, 2018) [40]
* Bifidobacteriales *	Significant perturbation (Casero D, 2017) [26]
* Coriobacteriales *	Significant perturbation (Casero D, 2017) [26]
* Verrucomicrobiales *	Significant perturbation (Casero D, 2017) [26]
* Lactobacillales *	Significant perturbation (Casero D, 2017) [26]
* Bacteroidales *	Increased (Lam Vy, 2012) [37]
Family	
* Desulfovibrionaceae *	Increased (Li Y, 2020) [17] Increased (Lavrinienko A, 2020) [39] Some members tolerate high radiation levels (Lavrinienko A, 2018) [38]
* Staphylococcaceae *	Increased (Goudarzi M, 2016) [19]
* Lactobacillacea *	Increased (Goudarzi M, 2016) [19] Decreased (Li Y, 2020) [17]
*Prevotellacea*	Unaltered (Lu L, 2019) [25]
*Clostridiaceae*	Irradiation-resistant bacteria (Sittipo P, 2020) [21] Decreased (Goudarzi M, 2016) [19] Clostridiaceae_1—increased *p* = 0.042 (Zhao Y, 2019) [31] Increased (Cai Z, 2018) [43] Unchanged abundance. 47 separate *Clostridiaceae* OTUs with decreased expression (Lam Vy, 2012) [37]
*Lachnospiracea*	Irradiation-resistant bacteria (Sittipo P, 2020) [21] Decreased (Lu L, 2019) [25] Decreased (Goudarzi M, 2016) [19] Increased (Liu X, 2019) [23] Increased (Tong, 2022) [34] Increased (Lavrinienko A, 2020) [39] Significant changes 1 week post-radiation compared with unirradiated group (*p* <0.05). Largely reversed in chronic phase (*p* < 0.05) (Li Yiyi, 2020) [32] Lachnospiraceae_NK4A136—Decreased (Wang M, 2020) [28] uncultured_bacterium_f_Lachnospiraceae—decreased (Wang M, 2020) [28] Minipigs—unclassified Lachnospiraceae—significantly decreased. Macaques—Increased(Carbonero F, 2018) [40]
*Moraxellaceae*	Decreased (Tong, 2022) [34]
*Ruminococcaceae*	Irradiation-resistant bacteria (Sittipo P, 2020) [21] Decreased (Lu L, 2019) [25] Decreased. Two members of the Ruminococcaceae family increased (Goudarzi M, 2016) [19] Increased in the LT10 group (Liu X, 2019) [23] Increase (*p* < 0.05) (Lavrinienko A, 2020) [39]
*Porphyromonadaceae*	Significant decrease (Liu X, 2019) [23]
*Rikenellaceae*	Significant changes in 1 week post-radiation (*p* < 0.05). Largely reversed in chronic phase of the disease (*p* < 0.05) (Li Yiyi, 2020) [32]
*Eggerthellaceae*	Significant changes in 1 week post-radiation (*p* < 0.05). (Li Yiyi, 2020) [32]
*Enterobacteriaceae*	Decreased 2 h after and significantly decreased 16 h after *p* < 0.05. No significant differences 24 h after. (Johnson, 2004) [24] Decreased (Tong, 2022) [34] Decreased (Cai Z, 2018) [43]
*Flavobacteriaceae*	Increased significantly (Cai Z, 2018) [43]
*Muribaculaceae* *S24-7 family*	Significant changes in 1 week post-radiation (*p* < 0.05). Largely reversed in chronic phase (*p* < 0.05) (Li Yiyi, 2020) [32] Bacteroidales_S24-7_group increased (Wang W, 2020) [30] uncultured_ bacterium_f_Bacteroidales_S24-7_group –increased 30 days after (Wang W, 2020) [30] Enrichment of members. In second capture—CL: decrease in abundance of members (>10% reduction) (Lavrinienko A, 2020) [39]
*Bacillaceae*	Increased significantly (Cai Z, 2018) [43]
*Xanthomonadaceae*	Increased significantly (Cai Z, 2018) [43]
*Sphingobacteriaceae*	Increased significantly (Cai Z, 2018) [43]
*Aeromonadacea*	Increased significantly (Cai Z, 2018) [43]
*Peptostreptococcaceae*	Unchanged (Lam Vy, 2012) [37]
*Veillonellaceae*	Macaques—Increased (Carbonero F, 2018) [40]
Genus	
*Acinetobacter*	Decreased (Kalkeri, 2021) [42]
*Aerococcus*	Decreased (Kalkeri, 2021) [42]
*Actinobacillus*	Significantly increased (Kalkeri, 2021) [42]
*Actinobacteria major genera*	Decreased (Carbonero F, 2018) [41]
*Akkermansia*	Increased (Kim YS, 2015) [27] Significant change in abundance. (Gerassy-Vainberg, 2018) [22] Increased (Wang W, 2020) [30] Significantly increased (*p* < 0.05). (Li Yiyi, 2020) [32]
*Alloprevotella*	Decreased (Wang W, 2020) [30] Increased (Cheema, 2021) [35] Significant changes in 1 week (*p* < 0.05). Largely reversed in chronic phase (*p* < 0.05) (Li Yiyi, 2020) [32]
*Alistipes*	Decreased (Lu L, 2019) [25] Increased in large intestine (>5%). Small intestine—decreased (Kim YS, 2015) [27] Increased (Wang M, 2020) [28] Increased (Carbonero F, 2018) [41] Increased 7 days after and reversed 30 days after (Wang W, 2020) [30] Significant changes in 1 week post-radiation (*p* < 0.05). Largely reversed in chronic phase (*p* < 0.05) (Li Yiyi, 2020) [32]
*Anaerotruncus*	Increased (Li Y, 2020) [17]
*Bacteroides*	Decreased (Kim YS, 2015) [27] Increased (Wang M, 2020) [28] Increased (Cheema, 2021) [35] Decreased LT10 group (Liu X, 2019) [23] Decreased—(Wang W, 2020) [30] Significant changes in 1 week post-radiation (*p* < 0.05). Largely reversed in chronic phase (*p* < 0.05) (Li Yiyi, 2020) [32] Significantly increased (Kalkeri, 2021) [42] Minipigs—decreased (Carbonero F, 2018) [40] Minipigs—All radiation levels led to significant decreases (Carbonero F, 2018) [41] Macaques—High radiation levels—increase (Carbonero F, 2018) [41]
*Barnesiella*	Decreased (Kim YS, 2015) [27] Decreased (Liu X, 2019) [23]
*Betaproteobacteria members (Desulfovibrio and Bilophila)*	Macaques—Irradiation at all levels significantly decreases/At day 3 were also increased at all radiation levels (Carbonero F, 2018) [41]
*Bacillus* spp.	Decreased (Raber J, 2020) [33] Decreased (Ben Ami, 2020) [44]
*Bifidobacterium*	Decreased in 2. However, in the 4 Gy–irradiated group increased ~10 times after 48 h and reached 28 times after 72 h. (Yamanouchi K, 2019) [18] Decreased (Carbonero F, 2018) [41] Decreased 30 days after exposure compared to their 10-day (Casero D, 2017) [26]
*Butyricimonas*	Decreased (Carbonero F, 2018) [41]
*Blautia*	Minipigs and Macaques—increases (Carbonero F, 2018) [41] Minipigs—Increased (Carbonero F, 2018) [40]
*Citrobacter* sp.	Decreased (Ben Ami, 2020) [44]
*Collinsella*	Decreased (Carbonero F, 2018) [41]
*Coprococcus_1*	Increased (Li Y, 2020) [17]
*Corynebacterium*	Increase (Kim YS, 2015) [27]
*Clostridium*	Significantly increased in a time-dependent manner (Lu L, 2019) [25] Minipigs—All radiation—Significant decreases; Macaques—increases in High levels (Carbonero F, 2018) [41]
*Clostridium cluster IV, XIVa* and *XIVb*	Minipigs and Macaques—High radiation level increases (Carbonero F, 2018) [41] Clostridium XIVa Significant positive correlations (Carbonero F, 2018) [41]
*Dubosiella*	Significant changes in 1 week post-radiation (*p* < 0.05). Largely reversed in chronic phase (*p* < 0.05) (Li Yiyi, 2020) [32]
*Elusimicrobium*	Significant decreases (Carbonero F, 2018) [41]
*Enterobacter* sp.	Increased (Ben Ami, 2020) [44]
*Enterococcus*	Significant changes in 1 week post-radiation (*p* < 0.05). Largely reversed in chronic phase (*p* < 0.05) (Li Yiyi, 2020) [32]
*Escherichia-Shigella*	Increased (Lu L, 2019) [25] Significant changes in 1 week post-radiation (*p* < 0.05). Largely reversed in chronic phase (*p* < 0.05) (Li Yiyi, 2020) [32]
*Eubacterium*_coprostanoligenes_group	Decreased (Wang W, 2020) [30]
*Faecalibacterium*	Decreases (Carbonero F, 2018) [41]
*Helicobacter*	Significantly increased (Lu L, 2019) [25] Decreased (Carbonero F, 2018) [41] Minipigs and Macaques—Increased (Carbonero F, 2018) [40]
*Klebsiella* sp.	Decreased (Ben Ami, 2020) [44]
*Lactobacillus*	No significant changes (Lu L, 2019) [25] Decreased D1, recovered at D3 and D10 (Sittipo P, 2020) [21] Decreased from 6 h to 12 h and then recovered to baseline. (Yamanouchi K, 2019) [18] Increased in the large intestine (>5%) (Kim YS, 2015) [27] Decreased (Wang M, 2020) [28] Decreased 2 h after and significant decrease after sixteen *p* < 0.05. No significant differences 24 h after. (Johnson, 2004) [24] Decreased (Cheema, 2021) [35] Significantly increased (*p* < 0.05) (Li Yiyi, 2020) [32] Minipigs—Increased (Carbonero F, 2018) [40] Minipigs—Correlate negatively; Macaques—sharp increase of only immediately after irradiation/correlate negatively (Carbonero F, 2018) [41]
*Mucispirilum*	Decreased (Kim YS, 2015) [27]
*Olsenella*	Increases High radiation levels (Carbonero F, 2018) [41]
*Oscillibacter*	Decreased (Kim YS, 2015) [27] Significantly increased (Lu L, 2019) [25] Minipigs—Increased (Carbonero F, 2018) [40]
*Parabacteroides*	Increased (Wang M, 2020) [28] Increased (Carbonero F, 2018) [41]
*Paraprevotella*	Macaques—Increased (Carbonero F, 2018) [41] Minipigs—Decreased (Carbonero F, 2018) [40]
*Pseudomonas* sp.	Increased (Ben Ami, 2020) [44]
*Pseudoflavonifractor*	Reduced the proportions (Kim YS, 2015) [27]
*Prevotella*	Decreased (Kim YS, 2015) [27] Minipigs and Macaques—significantly decreased (Carbonero F, 2018) [41] Significantly increased (Kalkeri, 2021) [42]
*Providencia* sp.	Decreased (Ben Ami, 2020) [44]
*Quinella*	Decreased significantly; *p* = 0.029 (Zhao Y, 2019) [31]
*Ralstonia* sp.	Decreased (Ben Ami, 2020) [44]
*Roseburia*	Decreased (Lu L, 2019) [25] Increased (Casero D, 2017) [26] Minipigs—Significant decreased (Carbonero F, 2018) [40]
*Ruminococcus*	Significant positive correlations (Carbonero F, 2018) [41] Minipigs—Significant decreased (Carbonero F, 2018) [40]
*Slackia*	Macaques—decrease higher radiation levels (Carbonero F, 2018) [41]
*Streptococcus*	Minipigs: Significant increased/Macaques: Sharp decrease (Carbonero F, 2018) [41] Minipigs—Increased (Carbonero F, 2018) [40]
*Suterella* spp.	Significant change (Gerassy-Vainberg, 2018) [22]
*Treponema*	Macaques—significant decreases in all radiation levels/Higher radiation levels induced immediate increase. On day 3, members increased at all radiation levels (Carbonero F, 2018) [41] Macaques and Minipigs—Increased (Carbonero F, 2018) [40]
*Veillonella*	Significantly increased (Kalkeri, 2021) [42]
Species	
*Adlercreutzia unclassified*	Decrease 30 days after (Casero D, 2017)[26]
* Akkermansia muciniphila *	Increase (Casero D, 2017) [26]
* Clostridiaceae * species	Decrease 30 days after (Casero D, 2017) [26]
*Eubacterium biforme*	Decrease (Goudarzi M, 2016) [19]
* Mollicutes * species (*Tenericutes* phylum)	Extinguish after exposure to 0.25 Gy of 16O (Casero D, 2017) [26]
*Prevotellaceae_UCG-001*	Decreased (Wang M, 2020) [28] Decreased (Wang W, 2020) [30]
*Ruminococcaceae_UCG-014*	Increased relative abundance (Wang M, 2020) [28] Increased 7 days after and decreased 30 days after (Wang W, 2020) [30]
* Ruminococcus gnavus *	Declined significantly (Goudarzi M, 2016) [19] Increased (Casero D, 2017) [26]
* S24–7 * unclassified species	Decrease 30 days after exposure (Casero D, 2017) [26]
Unclassified *Lactobacillus*	Decrease 30 days after (Casero D, 2017) [34]
*uncultured_bacterium_g_Acinetobacter*,	Decreased (Tong, 2022) [34]
*uncultured_bacterium_o_, Mollicutes_RF39*,	Decreased (Tong, 2022) [34]
*uncultured_bacterium_g_Citrobacter*,	Decreased (Tong, 2022) [34]
*uncultured_bacterium_g_Lactococcus—decreased*	Decreased (Tong, 2022) [34]
*Streptococcus_gallolyticus*	Decrease significantly; *p* = 0.034 (Zhao Y, 2019) [31]

## Data Availability

Not applicable.

## Supplementary Materials

The following supporting information can be downloaded at https://www.mdpi.com/article/10.3390/cimb45050249/s1, Table S1—Risk of Bias of the analyzed interventional studies.
