# Supplementary material for: The Effects of Ionizing Radiation on Gut Microbiota: What Can Animal Models Tell Us?—A Systematic Review
Source: Curr Issues Mol Biol. 2023 May 2;45(5):3877–910. doi: 10.3390/cimb45050249 (PMC10217073; doi:10.3390/cimb45050249)
Supplement: Supplementary file 1 [file cimb-45-00249-s001.zip › cimb-2312320-supplementary.pdf]

Table S1 – Risk of Bias of the analyzed interventional studies

	Selection bias			Performance bias		Detection bias		Attrition bias	Reporting bias	Other
	Sequence generation	Baseline characteristics	Allocation concealment	Random housing	Blinding	Random outcome assessment	Blinding	Incomplete outcome data	Selective outcome reporting	Other sources of bias
Kalkeri, 2021[42]	Unclear	Yes	Unclear	Yes	No	No	Yes	Unclear	Yes	Yes
Tong JY, 2022[34]	Unclear	Yes	Unclear	Yes	No	No	Yes	Unclear	Yes	Yes
Cheema AK, 2021[35]	Unclear	Yes	Unclear	Yes	No	No	Yes	Unclear	Yes	Yes
Li Y, 2020 [17]	Unclear	Yes	Unclear	Yes	No	No	Yes	No	Yes	Yes
(Johnson, 2004) [24]	Unclear	Yes	Unclear	Yes	No	No	Yes	Unclear	Yes	Yes
Sittipo P, 2020 [21]	Unclear	Yes	Unclear	Yes	No	No	Yes	Unclear	Yes	Yes
Raber J, 2020 [33]	Unclear	Yes	Unclear	Yes	No	No	Yes	Yes	Unclear	Yes
Cai Z, 2018 [43]	Unclear	Yes	Unclear	Yes	No	No	Yes	Yes	Yes	Yes
Rentea RM, 2016 [36]	Unclear	Yes	Unclear	Yes	No	No	Yes	Unclear	Yes	Yes
Lam Vy, 2012 [37]	Unclear	Yes	Unclear	Yes	No	No	Yes	Yes	Yes	Yes
Cui M,	Unclear	Yes	Unclear	Yes	No	No	Yes	No	Yes	Yes

2017 [20]										
Carbonero F, 2018 [41]	Unclear	Yes	Unclear	Yes	No	No	Yes	Unclear	Yes	Yes
(Carbonero F, 2018) [40]	Unclear	Yes	Unclear	Yes	No	No	Yes	Unclear	Yes	Yes
(Casero D, 2017)[26]	Unclear	Yes	Unclear	Yes	No	No	Yes	Unclear	Yes	Yes
Ben Ami, 2020 [44]	Unclear	Yes	Unclear	Yes	No	No	Yes	Unclear	Yes	Yes
Wang M, 2020 [28]	Unclear	Yes	Unclear	Yes	No	No	Yes	Unclear	Yes	Yes
Wang W, 2020 [30]	Unclear	No	Unclear	Yes	No	No	Yes	Unclear	Yes	Yes
Zhao Y, 2019) [31]	Unclear	Yes	Unclear	Yes	No	No	Yes	Unclear	Yes	Yes
Zhao Z, 2020 [29]	Unclear	Yes	Unclear	Yes	No	No	Yes	Unclear	Yes	Yes
Yamanouchi K, 2019 [18]	Unclear	Yes	Unclear	Yes	No	No	Yes	Unclear	Yes	Yes
Woruba DN, 2019 [45]	Unclear	Yes	Unclear	Yes	No	No	Yes	Unclear	Yes	Yes
Goudarzi M, 2016 [19]	Unclear	Yes	Unclear	Yes	No	No	Yes	Unclear	Yes	Yes
Kim YS, 2015 [27]	Unclear	Yes	Unclear	Yes	No	No	Yes	Unclear	Yes	Yes

Gerassy-Vainberg, 2018 [22]	Unclear	Yes	Unclear	Yes	No	No	Yes	Unclear	Yes	Yes
Liu X, 2019 [23]	Unclear	Yes	Unclear	Yes	No	No	Yes	Yes	Yes	Yes
Lu L, 2020 [25]	Unclear	Yes	Unclear	Yes	No	No	Yes	Yes	Yes	Yes
